# The value of co-creating a clinical outcome assessment strategy for clinical trial research: process and lessons learnt

**DOI:** 10.1186/s40900-023-00505-7

**Published:** 2023-10-24

**Authors:** Thomas Morel, Karlin Schroeder, Sophie Cleanthous, John Andrejack, Geraldine Blavat, William Brooks, Lesley Gosden, Carroll Siu, Natasha Ratcliffe, Ashley F. Slagle

**Affiliations:** 1 Patient-Centred Outcomes Research, UCB Pharma, Allée de La Recherche 60, 1070 Anderlecht, Brussels, Belgium; 2 Parkinson’s Foundation, New York, NY USA; 3 Modus Outcomes, a Division of Thread, London, UK; 4 Parkinson’s Foundation, New York, NY USA; 5 Parkinson’s UK, London, UK; 6 Aspen Consulting, LLC, Steamboat Springs, CO USA

**Keywords:** Early-stage Parkinson’s, Parkinson’s disease, Patient-reported outcomes, Patient expert, Patient engagement, Co-creation, Patient and public involvement, Patient-focused drug development, Outcome measure, Clinical outcome assessment, PEIRS-22

## Abstract

**Background:**

In support of UCB pharmaceutical research programs, the aim of this research was to implement a novel process for patient involvement in a multidisciplinary research group to co-create a clinical outcome assessment strategy to accurately reflect the experience of people living with early-stage Parkinson’s. Patient experts were an integral part of the decision-making process for patient-reported outcome (PRO) research and instrument development.

**Methods:**

In partnership with two patient organizations (Parkinson’s UK and the Parkinson’s Foundation), 6 patient experts were recruited into a multidisciplinary research group alongside clinical, patient engagement and involvement, regulatory science, and outcome measurement experts. The group was involved across two phases of research; the first phase identified what symptoms are cardinal to the experience of living with early-stage Parkinson’s and the second phase involved the development of PRO instruments to better assess the symptoms that are important to people living with early-stage Parkinson’s. Patient experts were important in performing a variety of roles, in particular, qualitative study protocol design, conceptual model development, and subsequent co-creation of two PRO instruments.

**Results:**

Involving people with Parkinson’s in PRO research ensured that the expertise of these representatives from the Parkinson’s community shaped and drove the research; as such, PRO instruments were being developed with the patient at the forefront. Working with patient experts required considerable resource and time allocation for planning, communication, document development, and organizing meetings; however, their input enriched the development of PRO instruments and was vital in developing PRO instruments that are more meaningful for people with Parkinson’s and clinicians.

**Conclusions:**

Conducting PRO research, in the context of clinical development involving pharmaceutical companies, requires balancing regulatory and scientific rigor with tight time constraints. Incorporating a multi-stakeholder perspective, which included patient experts as joint investigators, had a strong positive impact on our research, despite the logistical complexities of their involvement. Due to the input of patient experts, the innovative clinical outcome assessment strategy and the co-created novel PRO instruments were more relevant and holistic to the patient experience of early-stage Parkinson’s.

**Graphical abstract:**

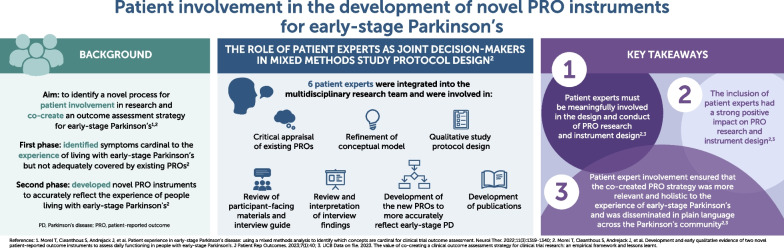

**Supplementary Information:**

The online version contains supplementary material available at 10.1186/s40900-023-00505-7.

## Abstract (plain language summary)

Patient-reported outcome (PRO) instruments allow people living with a disease and participating in a clinical study to describe the symptoms and experiences that they consider meaningful. PRO instruments use tools such as questionnaires and scales to capture patient perspectives on a treatment that might not be captured by a clinical measurement. It is recommended that the patient community and patient experts are included in the development of PRO instruments to accurately capture information that is important to them. Building on the experience of a recent PRO research project in support of UCB pharmaceutical programs, this article provides recommendations on how pharmaceutical companies can partner with patient organizations and involve patient experts as joint investigators in the co creation of PRO instruments. Despite the additional resource and time required, involving patient experts and patient organizations into the research collaboration had a strong positive impact and ensured that the PROs were meaningful to patients (in this instance, people living with early-stage Parkinson’s). Patient organizations facilitated patient engagement and recruitment in research activities, maintained communication with the pharmaceutical company’s research team, and built trust between collaborators by implementing patient engagement tools and best practices. Patient experts contributed to several parts of the PRO instrument development process: study design, identifying key symptoms and experiences, and developing individual PRO questions. Co-creation between the pharmaceutical company, patient experts, and patient organizations resulted in considerable improvements to typical PRO instrument development for use in clinical trials and is thus recommended.

**Supplementary Information:**

The online version contains supplementary material available at 10.1186/s40900-023-00505-7.

## Background

The inclusion of patients into clinical outcomes assessment (COA) development teams is inconsistent and not yet routine, despite initiatives promoting patient involvement in drug development such as the European Patients’ Academy on Therapeutic Innovation (EUPATI, Europe), Patient Focused Medicines Development (PFMD, Europe), Innovative Medicines Initiative (IMI) PARADIGM (Europe), National Health Council (NHC, USA), and Patient-Focused Drug Development (PFDD, Europe) guidance from the Food and Drug Administration (FDA, USA) and International Council on Harmonization (ICH) [1–7]. Existing examples of patient involvement in COA [8–11] are mostly in the context of academia or academic societies. Co-creation of patient-reported outcome (PRO) instruments–a type of COA–to more accurately reflect the lived-experience of patients is recommended [12] and requires specific types of evidence [13].

To effectively gather evidence, the pharmaceutical company (UCB) has focused on integrating patients into decision-making for COA development within the context of the pharmaceutical industry research and development (R&D) environment. Our previously published COA research [14–16] involved a multidisciplinary research group established in 2018, which comprised the following experts: clinical, patient-centered outcomes research (PCOR), patient engagement and involvement (from two patient organizations [POs]: Parkinson’s UK and the Parkinson’s Foundation), regulatory science, outcome measurement, movement disorder neurologists, and people living with Parkinson’s who were recruited via the two POs. This article aims to provide detailed insight into the implementation of the novel process for patient involvement that was used to co-create a COA strategy that more accurately reflects the lived-experience of early-stage Parkinson’s, and present learnings for future research. The article describes the planning and organizational stages necessary for co-creation, roles performed by patient experts in the multidisciplinary research group throughout the project, and areas where patient experts can provide an impact and value to the output.

## Methods

A two-phase research study was devised. The first phase aimed to identify what symptoms are cardinal to the experience of living with early-stage Parkinson’s and the second phase involved the development of PRO instruments to better assess the symptoms that are important to people living with early-stage Parkinson’s. Input from and collaboration with the Parkinson’s patient community was fundamental to the successful conduct of both phases of the research, and to support the overall goal of co-creation of PRO instruments that are relevant to the patients’ experience of early-stage Parkinson’s. Here we describe our approach to engagement and collaboration with patient experts, POs, and people living with early-stage Parkinson’s, and the range of roles they fulfilled in this collaborative research project.

### Planning patient engagement

#### Setting up for co-creation

Collaboration with POs facilitates patient engagement in research activities, including supporting patient involvement in the research process, maintaining communication with the pharmaceutical company’s research team, and building trust between collaborators. POs also introduce and pilot patient engagement tools, resources, best practices, and metrics, while overseeing critical tactical operations such as recruitment for patient interviews. The pharmaceutical company has collaborated with the Parkinson’s Foundation (since 2015) and Parkinson’s UK (since 2017), on several patient engagement projects, which prepared the organizations for co-creation of a COA strategy for early-stage Parkinson’s. The POs were important to this stage, as they were able to leverage their history with patients as well as their experience with and contribution to the Parkinson’s community, which allowed them to share valuable insight on how best to engage with patients and deliver a successful co-creation experience.

Firstly, principles of collaboration were agreed, and calls were organized to formally brief respective parties on research expectations, overall fit into R&D processes, and timelines. The long-term nature of the R&D process and where/how COA research fits into overall R&D plans were explained to the POs. Historically, patient expertise has not been properly acknowledged by the pharmaceutical industry, and so it was important to establish trust and ensure genuine commitment to meaningful patient engagement. Hence, the pharmaceutical company was made aware of the importance of the unique knowledge contribution of patient experts and timely sharing of results with the broader patient and scientific communities. Mutual understanding and alignment of POs and the pharmaceutical company at project inception were established to enable a working relationship built on trust and ensure later success.

#### Legal and compliance considerations

Legal and compliance agreements were complex and newly developed for this project, taking 4–6 months to complete. A master collaboration agreement between the pharmaceutical company and the POs was developed in 2018, with the core principle of co-creation of a patient-relevant COA strategy for clinical trials in early-stage Parkinson’s. To minimize workload and legal input, the contract was as succinct as possible, covering key issues of confidentiality, intellectual property, copyright, data protection, compensation, and responsibilities. Its structure reflected the Guiding Principles for Legal Agreements later published in October 2020 by IMI PARADIGM [17]. Contract items requiring greater time and attention included: joint Intellectual Property ownership, financial compensation for patients involved in the research team and their representatives, commitment to timelines for external communications and the development of peer-reviewed material, and standards for working ethics and patient feedback communications. Due to the imbalance in legal resources between the pharmaceutical company and POs, the legal process was kept as simple as possible, whilst including clear definitions of the different roles and maintaining full transparency on the risks and benefits of partnership for each party.

#### Diversity in patient expert representation

The POs were well-established with robust patient involvement/engagement networks that included people with a broad range of Parkinson’s experience. To recruit patients to join the multidisciplinary team, the Parkinson’s Foundation reached out to their network of Research Advocates; a group of ~ 200 people who have Parkinson’s or are caregivers who are trained to collaborate with research teams on the design and implementation of studies. Parkinson’s UK contacted their network of patient and public involvement contributors; a network of  ~ 160 people with Parkinson’s, partners, family members, and caregivers who had received introductory training on patient involvement in research. These networks enabled the pharmaceutical company and POs to recruit 6 people with Parkinson’s (3 each from the UK and the US for an international perspective), referred to as ‘patient experts’ because of their first-hand experience of Parkinson’s symptoms and the impact of the condition on all aspects of their lives [18]. It is important to note that the 6 patient experts differ from the 50 patient interviewees and the 60 patient interviewees in the two-phase research study; the patient experts worked in collaboration with UCB and the POs as equal research partners [19], whereas the two other groups of patient interviewees were the patients about whom key qualitative information was collected for the purposes of the research. The latter were not involved in the development of the COA.

Patient experts were selected for a wide range of Parkinson’s experiences. Although the research focus was early-stage Parkinson’s, it was important to consider patient experts with experience of Parkinson’s research, including participation in clinical trials, and those with early- and late-stage Parkinson’s to provide more context to the research and COA development activities. The races of the group were white (n = 5) and Asian (Chinese) (n = 1), with an equal split in terms of gender and geographic location (i.e., UK and US). The choice of 6 patient experts was a pragmatic one as this number enabled the research group to fully support their involvement and to ensure everyone’s voices could be heard during meetings and all opinions reflected in the feedback. In addition, while we wanted to involve experts with a diverse range of experiences and backgrounds, it would be difficult for a group of patient experts working in this capacity to be fully representative of such a wide patient group. Instead, their role was to advise based on their lived experience, their knowledge of the Parkinson’s patient community, and their experience of being within this community and knowing other people affected by Parkinson’s in their respective countries.

The patient experts were equal research partners. In the same way other experts involved in the research group (e.g., clinicians) they were not asked to provide any evidence of their ability to contribute to the work (other than affiliations and self-reported practice patterns) and to be a representative of their respective professional backgrounds. Similarly, we did not require confirmed diagnosis from the patient experts and relied on their active participation and PO membership, as well as self-reported experiences of Parkinson’s disease (PD). This afforded equality within the research group, and supported the key value for the patient experts’ involvement in the research of being able to represent their community and its variety of voices. This is in contrast to individual patients who may or may not be affiliated with a PO, and whose expertise is more about their own lived experience with disease, as opposed to a community perspective.

#### Sharing roles and responsibilities

An overarching ‘shared purpose’ was defined upfront by the pharmaceutical company and POs and discussed with the patient experts. The aim was to ‘co-create’ a patient-relevant COA strategy intended for use in clinical trials in early-stage Parkinson’s, with patient experts actively involved in all stages of the research process, including setting the research agenda and analyzing data.

**Fig. 1:**
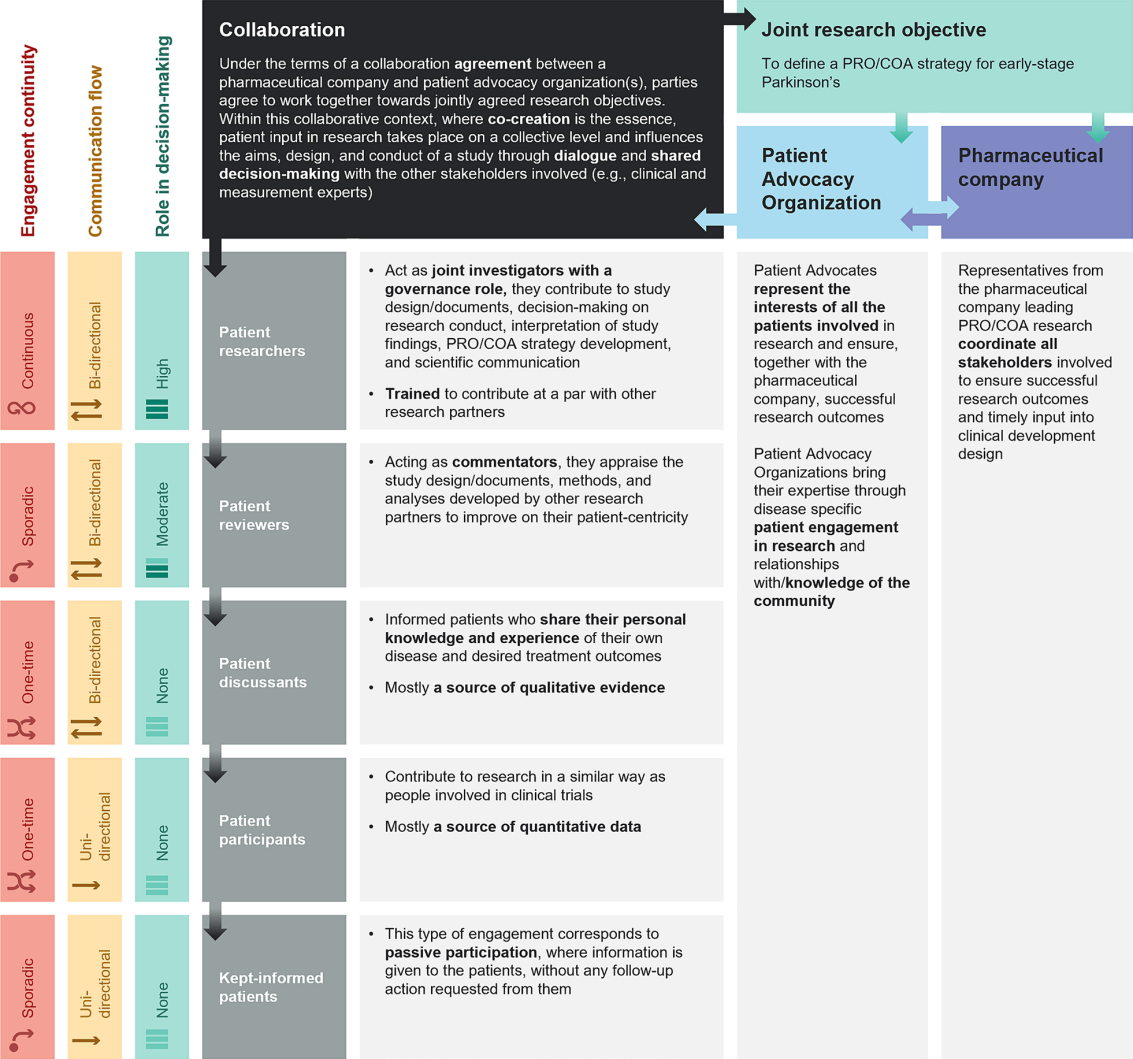
The Patient engagement model. This Patient engagement model, adapted from de Wit et al. [20] describes the levels of patient involvement throughout the development of a PRO strategy for early-stage Parkinson’s, ensuring that patient participation was meaningful at every stage of this process. *COA* clinical outcomes assessment, *PRO* patient-reported outcome

At project inception, a Patient Involvement Plan (PIP) was developed by all stakeholders and open debate was organized (via telephone) to ensure alignment. The PIP described who was involved and how, at each research stage, and achieved joint consensus and clarity on roles, responsibilities, and timelines, which was key to building and maintaining relationships. The PIP also described activity ownership, level of contribution, and expected outcomes, ensuring that the 6 patient experts were able to plan their contribution to the research. The introduction of the PIP is shown in Additional File 1. For the PIP, the ‘patient engagement ladder’ model from de Wit, et al. was adopted, which separates engagement into escalating levels: Inform, Consult, Advise, Collaborate, and Control [20]. This model was adapted by translating ‘action type’ (e.g., consultation, advise) into ‘patient role profile’ (e.g., patient participant, patient reviewer), and adding granularity to the ladder in terms of engagement continuity, communication flow, and role in decision-making (Fig. 1).

#### Accessibility and flexibility

Information and project materials (e.g., synthesis of study findings on mapped concepts; concept-to-item matrix to critically appraise PRO instruments) were made as accessible as possible to all team members, by avoiding jargon and terms unlikely to be understood by all team members, providing visuals and documents that were easy to navigate and respond to, and including probes (e.g., for feedback on draft conceptual models, cardinal concepts, or relevance of legacy instruments). Information was shared with patient experts verbally and in writing, with the opportunity for feedback in either format. Glossaries of research terminology were provided for presentations and ongoing reference.

Meeting materials and information were emailed at least 1 week in advance, with deadlines for feedback specified. Meeting times and locations were limited by geography; most occurred by web conference, with one face-to-face event. Consideration was given to the length of virtual and face-to-face meetings, as well as including breaks, and providing accessible overnight accommodation for patient experts for the face-to-face event. Where possible, meetings were recorded and summary notes circulated for those who could not attend.

UCB and the POs agreed to have the patient experts involved in select areas of decision-making, which were discussed with the patient experts and amended throughout the process to suit their individual needs. Patient experts were asked how much they wished to contribute to the project, at which timepoints, and in what manner, to accommodate their abilities with respect to their Parkinson’s and time periods they wished to commit to the work. They were also informed of the ways of being involved, which reflected professional experts’ opinions on where involvement of patient experts could be of most value. Each patient expert was provided with a role description outlining the areas in which their involvement was anticipated. This was discussed, agreed, and amended to ensure each patient expert’s role was suited to the commitment they were able to make. For the purposes of respect and availability, all stakeholders could communicate with each other at any time to build trust as the project evolved. Patient experts had dedicated contacts at their PO, and the pharmaceutical company and staff from the relevant PO worked together to coordinate all work.

### Conducting patient engagement in COA/PRO research

#### Building capacity to engage

During research planning it was acknowledged that relevant training on PRO instrument development would enable the patient experts to fully engage with the project and provide more widespread and insightful contributions.

A journal club provided background information on PRO instrument development and patient engagement (creating an opportunity for discussion on successful elements of COA/PRO instrument development and patient engagement work), the processes involved, and how they might inform the present study [21–24]. Stakeholders also viewed models of patient engagement from their different perspectives and discussed capacity for engagement, what was working well, and which areas could be improved.

A 3-h workshop on COA selection and development for use in clinical trials was presented for POs and patient expert stakeholders by PCOR and regulatory science experts to aid understanding of the process and regulatory needs/expectations.

#### Patient experts in phase I: study protocol, conceptual model development, and consensus on cardinal concepts

**Fig. 2:**
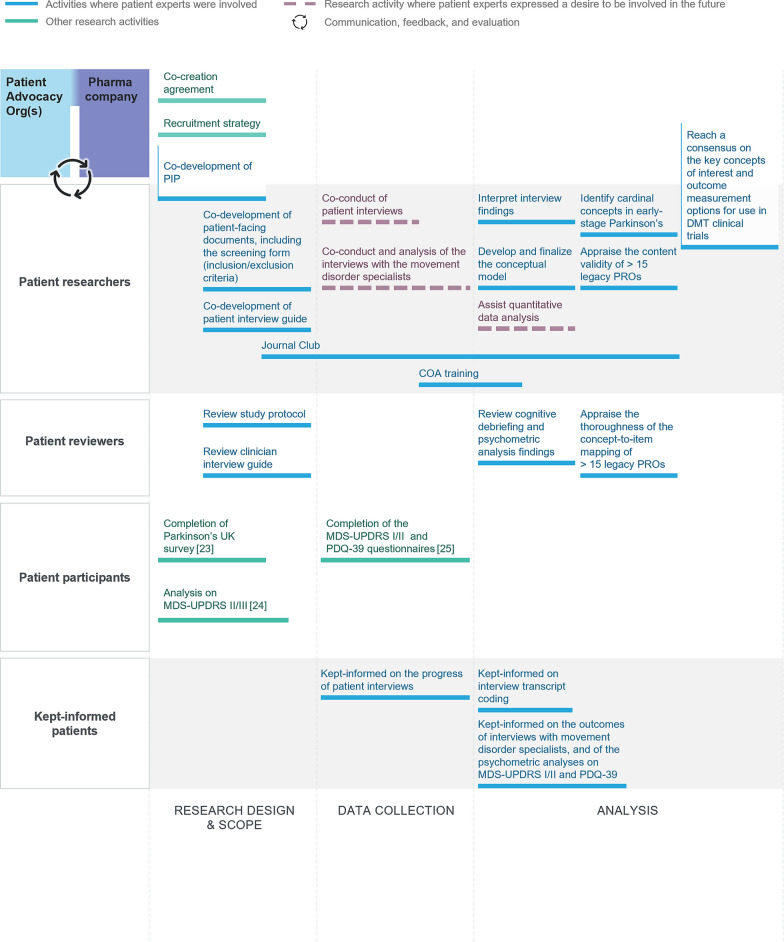
Patient roles for study design, identifying concepts of interest, and evaluating legacy clinical outcomes assessments. The figure displays the extent of patient contribution [26–28], which includes PRO research design, development of the conceptual model of the early-stage Parkinson’s experience, and appraisal of existing COAs in this context of use and identification of cardinal concepts. In addition to these patient roles, interviewees in phase I acted as ‘patient discussants’: 50 people living with early-stage Parkinson’s and 9 caregivers were interviewed to elicit concepts of interest and to appraise pre-existing PRO instruments, namely the MDS-UPDRS parts Ib and II and the PDQ-39. *COA* clinical outcomes assessment, *DMT* disease-modifying therapy, *PDQ-39* Parkinson’s Disease Questionnaire, *PIP* Patient Involvement Plan, *PRO* patient-reported outcome, *MDS-UPDRS* Movement Disorder Society-Unified Parkinson’s Disease Rating Scale

Conceptual models identify the relevant concepts (e.g., signs, symptoms, impacts) in a particular disease and show how they are thought to be inter-related, so that PRO instruments focus on the most important concepts for patients. Phase I of the research involved many different patient roles (Fig. 2) [25, 26], which led to mapping out and conceptualizing the important symptoms and experiences of people living with early-stage Parkinson’s, and reviewing how ‘fit-for-purpose’ the legacy PRO instruments were in measuring target concepts of interest [15, 27]. Cardinal concepts to be measured in trials evaluating disease-modifying therapies for Parkinson’s were also agreed on. Historically, patients have not participated in decision-making during this stage, but here, patient experts were involved throughout. They acted as ‘patient researchers’ for co-development of the PIP, screening form, and patient interview guide; for interpretation of interview findings and appraisal of legacy PRO instruments; and for development of the conceptual model, leading to the shortlist of cardinal concepts. Patient experts also acted as learned commentators (i.e., ‘patient reviewers’) [28], to review the draft study protocol, clinician interview guide, and cognitive debriefing findings.

Concept-to-item mapping research (to evaluate the relevance of existing legacy PRO instruments) was led by PCOR experts and critically reviewed by patient experts. After reviewing the skills required to contribute, a joint decision was taken that patient experts would not be involved in interviewing, qualitative coding, or psychometric analyses. Patient experts were kept informed during the patient and clinician interview, qualitative coding, and psychometric analysis stages.

Phase I found that legacy PRO instruments were of limited use for evaluating outcomes in early-stage Parkinson’s [27], indicating that new PRO instruments were required.

#### Patient experts in phase II: PRO instrument development

**Fig. 3:**
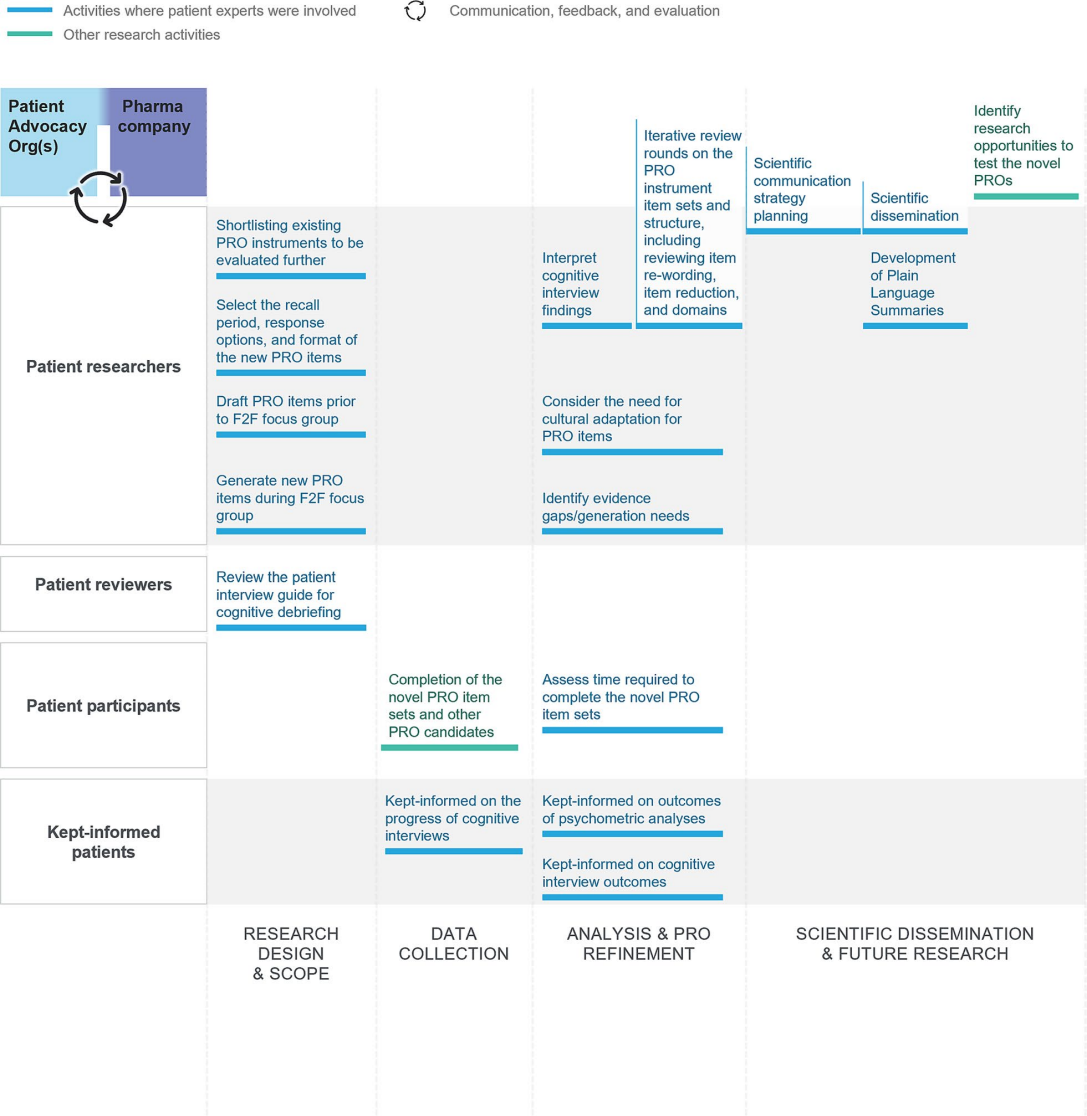
Patient roles in patient-reported outcomes development and scientific dissemination. The figure displays the levels of patient contribution to PRO instrument development iterations and scientific communications. In addition to these patient roles, interviewees in phase II acted as ‘patient discussants’: 60 people living with early-stage Parkinson’s were interviewed for cognitive debriefing activities on the preliminary PRO instrument item set and other PRO instruments. *F2F* face-to-face, *PRO* patient-reported outcome

Phase II of the research also included many different patient roles (Fig. 3), which led to the multidisciplinary research group developing fit-for-purpose PRO instruments to better assess what is important to patients with early-stage Parkinson’s. Patient experts collaborated on creation and iteration of the PRO instrument item sets and structure, instructions for patients (including language issues), and design and review of the cognitive debriefing documents and findings. Patient experts also contributed ideas for evidence generation needs and have been actively involved in developing scientific communications regarding the PRO research.

#### Interviewees in phase I and II

In phase I, 50 people with early-stage PD and 9 caregivers were interviewed (Fig. 2), acting as ‘patient discussants’ to elicit concepts of interest and to appraise pre-existing PRO instruments, namely the Movement Disorder Society-Unified Parkinson’s Disease Rating Scale (MDS-UPDRS) parts Ib and II and the Parkinson’s Disease Questionnaire (PDQ-39). In phase II, 60 people with early-stage PD were interviewed, as ‘discussants’, for cognitive debriefing activities on the preliminary PRO item set and other PRO instruments (Fig. 3). They also acted as ‘participants’ for completion of the draft PRO items. The patients who contributed to the Parkinson’s UK survey, whose output was used by our research team to triangulate our own research findings, were also considered study ‘participants’.

#### Assessing patient engagement

Formal surveys were conducted several times to check that patient experts were comfortable with their level of involvement and support. Bespoke surveys were circulated after certain key meetings and feedback was captured using PFMD’s Patient Engagement Quality Guidance Tool [29].

This research project was one of the first to implement the novel “Patient Engagement In Research Scale (PEIRS)” [30], to assess the degree of meaningful patient engagement in research over time. The final version, PEIRS-22, is a 22-item questionnaire made of 7 subscales: Procedural Requirements; Convenience; Contributions; Team Environment and Interaction; Support; Feel Valued; and Benefits [30]. As suggested by the authors, this survey, which was completed at the second year of research and at project closure, was used to monitor progress between PCOR researchers and patient experts to identify opportunities to improve working relationships.

## Results

### Patient expert feedback

Content from the journal club relating to PRO instruments was highlighted by patient experts as particularly useful for enhancing knowledge and encouraging positive contribution to the project. Representatives from the POs found that the journal club improved their support to patient experts and the project in general. Stakeholders viewing models of patient engagement also allowed for honest and open discussions and helped to build strong working relationships. All patient experts reported that the workshop on COA selection was very helpful. One patient expert commented that their confidence had grown as they gained a better understanding of the project as it progressed, which was helped by the enthusiasm and encouragement of the PCOR experts. It was important that knowledge continued to develop, and an encouraging and motivating environment was created as the project progressed. Overall, the training was not only beneficial for patient experts; it also helped PO representatives and research team members facilitate successful patient engagement.

Following the first phase of research, patient experts expressed that they would have liked to co-conduct concept elicitation interviewing activities and clinician interviews. These activities are usually performed by trained social scientists. Additional training for patient experts would be necessary to build the appropriate skills to participate in these activities [31].

### Evaluating the impact of co-creation in COA research

In their role as “patient researchers” (Fig. 1), the 6 patient experts made an important contribution to several parts of the development process, with considerable improvements to typical instrument development, including their content validity, for use in clinical trials and for regulatory purposes. The high level of patient expert engagement throughout ensured a positive impact on decision-making at all stages.

#### Areas of greatest impact of patient expert and PO involvement

##### Conceptualizing clinical benefit

The conceptual model of the early-stage Parkinson’s experience was developed iteratively via categorizing concepts identified during the phase I participant interviews into higher order domains. Patient experts played a critical role in streamlining concepts generated from the categorizations. Notably, the middle levels of the model were refined for a more granular representation of symptoms and impacts. For example, following patient experts’ advice, the concept of ‘freezing’ was moved from the bradykinesia symptom domain to the domains of activities affected by ‘freezing’ (i.e., mobility, speech, cognitive functioning). ‘Weakness’ and ‘lack of strength’ were also moved to the same domain. Patient expert review reduced the number of sub-domains under the ‘Psychological’ domain from 26 to 10. Patient expert input helped align the conceptual model closer to the patient experience, increasing the validity of the COA research output.

##### Direct disease experience

Patient experts offered continuous opportunities to discuss their disease experience to double-check the hypotheses, assumptions, or research conclusions made by the PCOR team members, and helped confirm the shortcomings of 15 legacy PRO instruments by validating conceptual content validity gaps. Patient experts also provided spontaneous feedback on the face validity of fatigue instruments to identify terms for further testing.

##### Identification and selection of cardinal concepts

Relying on the research conducted, as well as their experiences, patient experts helped identify the cardinal concepts that a disease-modifying therapy trial should focus on, namely: bradykinesia (particularly functional slowness), tremor, rigidity/stiffness, mobility (particularly fine motor dexterity and subtle gait abnormalities), and fatigue.

##### Decisions on content of newly generated items and overall COA strategy for the trial

Patient experts were instrumental in defining the item sets and crafting individual items to ensure the wording reflected the patients’ perspective and cultural/language differences. For example, patient experts identified that “walking outdoors” could be unclear depending on whether you are based in the UK or US and so, the item was updated to “difficulty walking on uneven ground” to add further context (see Additional file 2). This shaping of item content optimized its relevance, clarity, and appropriateness for use as a PRO instrument. Patient experts also helped shape the instructions to patients.

##### Enhancing the quality of study documentation

Patient experts ensured that the self-report screener for prospective interviewees was unambiguous and easy to complete, which was particularly important as there was no clinically confirmed diagnosis in this study. Furthermore, patient experts enhanced the adequacy of patient-facing documents (e.g., information sheets, consent forms) and helped improve the content of the interview guide. Specifically, building on the list of probes that were addressed to the interviewees further allowed the authors to elicit a content-rich set of concepts in relation to the patient experience and potential treatment benefit in early-stage Parkinson’s.

##### Opportunities for evidence generation

Patient experts flagged the importance of investigating the role of ‘laterality/handedness’ in Parkinson’s manifestations as a confounder of severity scoring–a request later communicated to the pharmaceutical company by regulatory agencies. POs’ own centers-of-excellence networks offered immediate opportunities to engage with leading clinicians and/or to access ongoing cohort studies to test the novel PRO instruments.

##### Impact on timelines

Incorporating POs and patient involvement in COA/PRO research naturally required additional time in the study plan. However, the pharmaceutical company–PO partnership enabled faster recruitment of study participants by providing access to pre-existing networks; over 250 relevant candidates for patient interviews responded within 10 days, and direct contacts with a network of clinical centers of excellence was available to organize clinician interviews.

### Challenges with patient involvement in COA research and the importance of continuous feedback and evaluation

The extent of patient involvement in this research project posed challenges as the research progressed, which are summarized, along with solutions, in Table 1. Regarding overall integration of patient experts into the research team, bi-directional feedback between them and the pharmaceutical company throughout the process and clear communication about the value of their input ensured that they felt respected and heard. Ensuring that patient experts remained informed and engaged was central to maximizing their involvement. Existing relationships with PO staff helped foster an open, honest environment for patient experts to share their thoughts, concerns, and suggestions; patient experts were also encouraged to ask for clarification at any time during meetings.

**Table 1:** Challenges and solutions for patient engagement in COA research

Challenge	Solutions and learnings
Capacity of patients to engage	Each research team member must balance their role with their own daily life and work priorities, and patients must also live with and manage their disease daily. Researchers should allow for this and be mindful of where agreed tasks exceed capacity or time constraints of patients via regular review
Mediation and communication	An orchestrator with good interpersonal skills to keep the team united and committed over time is vital, especially where patient experts disagreed with each other
Managing expectations and communication	Where patient advice is not taken forward, decisions must be explained transparently to retain trust and a sense of ownership/feeling valued; for example, not all patient suggestions for improvements or outcomes to measure may be feasible in a trial (either due to trial design, such as trial duration, or due to hypothesized treatment benefit resulting from treatment mode of action), may be easily translated into a study endpoint, or may be appropriate for approval and labeling decisions by regulators Maintenance of good communication on timelines and when patients may expect documents for comment may be challenging as the project progresses due to external factors including completion of the patient interviews or the volume of data to be analyzed Pharmaceutical companies should be prepared for the unique needs of the patient community partners. For in-person meetings patient partners experts may need to fly in a day early to avoid fatigue at the time of a meeting or stay at a hotel that is in a central location; this may lead to higher costs for meetings but is essential to address respect and accessibility
Managing sources of irritation	The long R&D timeframe may be frustrating to patients, especially if their disease is degenerative and disabling, and it is often the case that research to which they are contributing may not directly benefit them or the course of their personal disease experience; however, the patient experts in this research had regular experience of contributing to research that would not directly benefit them The duration of web-based meetings was challenging and required occasional in-depth analysis of data in a short time period, necessitating long calls. The maximum duration of calls was reduced from an initial 3 h and mid-way breaks were introduced, based on feedback from patient experts Time was an important project resource; all stakeholders had to respect meeting deadlines and the overall timescale for project plans. Unavoidable lengthy gaps between meetings and deadlines occasionally led to wavering motivation
Time and resource challenges	The imbalance in legal resources available to POs and the pharmaceutical company created delays in reaching mutually acceptable legal and compliance agreements Addressing training needs and preparing specific documents in lay terms (with probes, accessible visuals summarizing analysis outputs, etc.) to elicit patient expert feedback was also time and resource intensive Additional meetings to engage with patient experts required preparation of materials and post-meeting summary notes; some meetings had to be repeated to gather contributions from patient experts in different time zones Ensuring that technical aspects of the work were presented in a way that could be easily comprehensible by patient experts also required time to explain processes throughout The time required for proper publication development, with iterative rounds of review and shaping of content was not anticipated by patient experts and was a source of frustration, particularly where it was perceived that the patient-centered focus of the research was secondary to the tone of scientific reporting. With hindsight, better upfront communication of the publication process and timelines involved would have been beneficial. The inclusion of all 6 patient experts as co-authors also impacted progress, given the unpredictable nature of progressing Parkinson’s; future projects may benefit from appointing specific patients as co-authors to represent the group. However, having patient experts as co-authors, motivated the decision to publish all research outputs from project inception with plain language summaries

*COA* clinical outcomes assessment, *PO* patient organization, *R&D* research and development

**Fig. 4:**
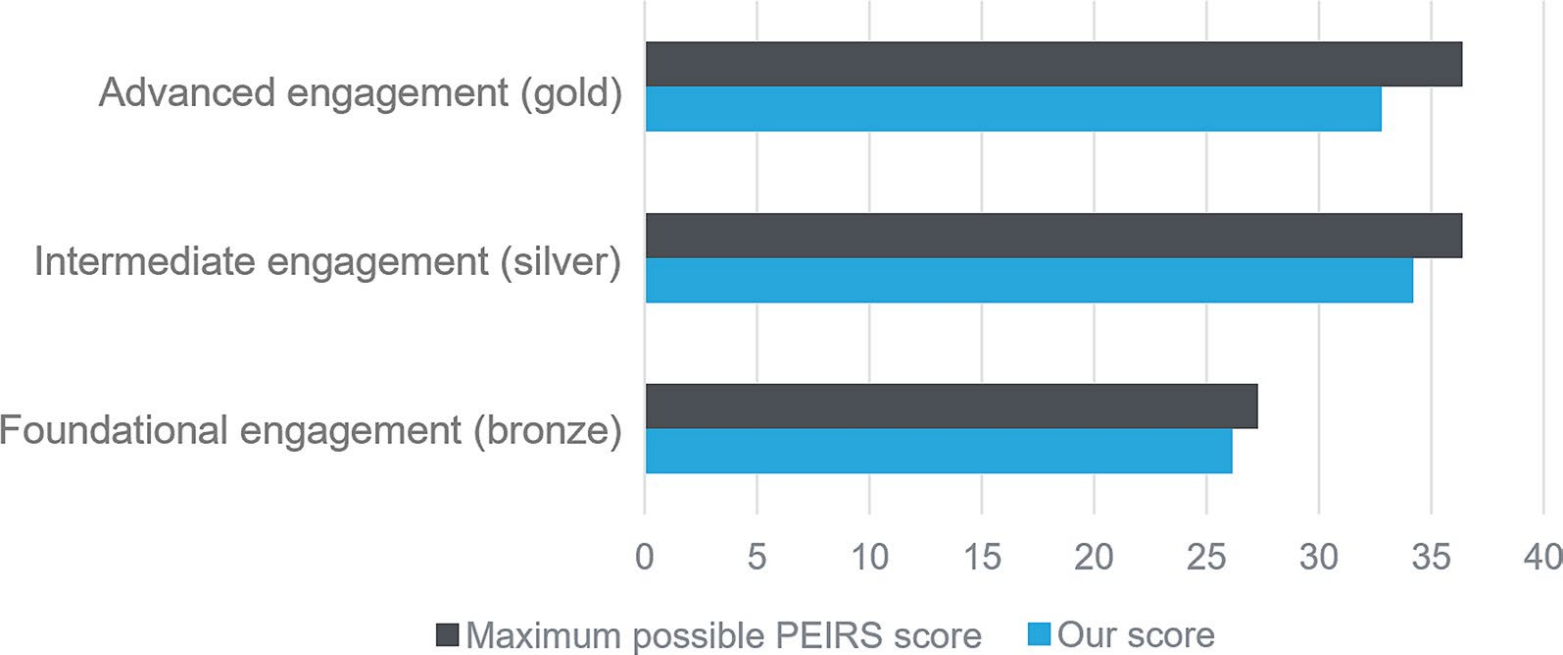
Patient Engagement In Research Scale (PEIRS) scores across phases I and II. PEIRS scores measure the degree of meaningful patient engagement across both stages of this COA research, from a patient perspective. The PEIRS-22 scoring manual [32] distributes the 22 items of the PEIRS-22 across three levels of meaningful engagement in research. Advanced engagement (gold level) is the most difficult to achieve as positive experiences of those important elements of meaningful engagement are least often reported by patient experts. Intermediate engagement (silver) and foundational engagement (bronze) reflect the aspects that are moderately and least difficult to achieve, respectively. *COA* clinical outcomes assessment, *PEIRS* Patient Engagement In Research Scale

In phases I and II, based on the PEIRS-22 scores, high meaningful engagement was achieved across all aspects (advanced, intermediate, foundational) from most to least difficult to achieve (Fig. 4) [30, 32]. Overall, the research project achieved a PEIRS-22 score of 93/100 (Additional file 3), which corresponds to a threshold of “extremely high meaningful engagement” [30, 32]. Furthermore, scores across the 7 dimensions of the PEIRS-22 were maintained or improved during the research (Additional file 3).

## Discussion

This article reports the co-creation of a PRO strategy for early-stage Parkinson’s with expanded roles for patient experts. Including patients in the research team provides a unique perspective based on first-hand experience of how Parkinson’s affects all aspects of patients’ lives, and how they feel about living with and managing their condition that cannot be replicated by anyone else [33]. This could contribute to the development of more effective patient-focused clinical trials [34]. Janet Woodcock, Director for the Center for Drug Evaluation and Research at the FDA at the time of this research, also emphasized the importance of the patient’s voice: “I cannot stress enough how important having the patient and the patient’s voice at the table is during drug development and in evaluating the safety and effectiveness of new medicines” [35]. Previously, patient involvement across medicines R&D has lacked structure and a consistent approach [36].

This project required balancing regulatory and scientific rigor (to make the PRO instruments suitable for use in randomized controlled trials) with the need for timely conduct of clinical trials to make new treatment options to patients as soon as possible, while incorporating a patient-predominant multi-stakeholder perspective. By establishing a strong patient engagement process to incorporate patient experts as joint investigators from the outset, a clear and robust plan suitable for all stakeholders was implemented. For this project, POs were highly valued partners that facilitated interactions between the patient experts and other members of the multidisciplinary research group. The Guidance for Reporting Involvement of Patients and the Public (Additional File 4) outlines how patient experts were involved in this project.

## Lessons for the future

**Fig. 5:**
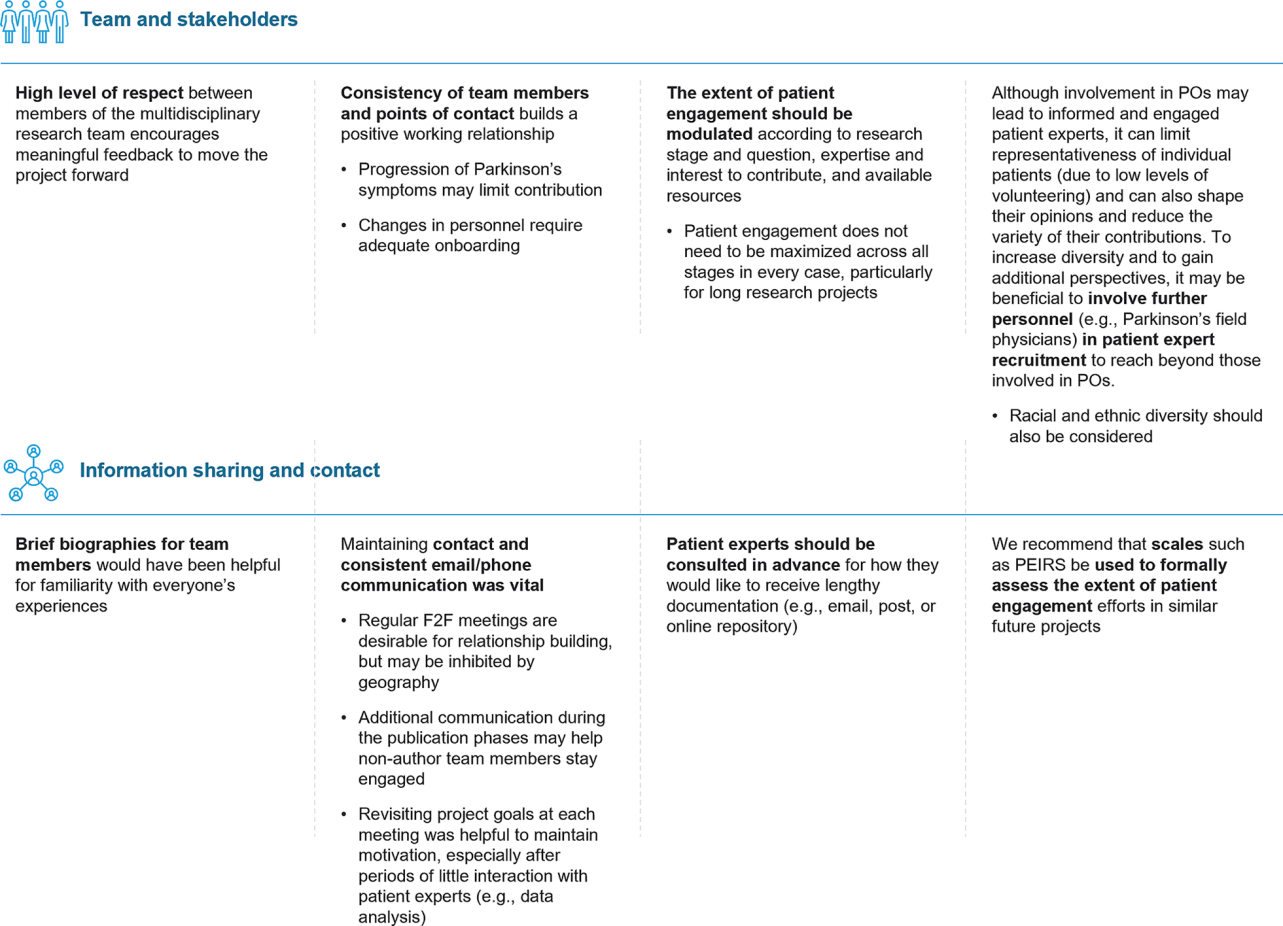
Lessons for the future. *F2F* face-to-face, *PO* patient organizations, *PEIRS* Patient Engagement In Research Scale

Learnings from this study are described in Fig. 5. Fortunately, all stakeholder team members were present for the duration of this work (over 3 years); this contributed to the positive working relationship of the group. However, in other studies, Parkinson’s progression could limit the input of patient experts despite the individual still wishing to be involved in the research. In this circumstance, it is important to consider shifting their role from a highly involved participant to an advisory capacity, and plan for this succession from the outset. Furthermore, for future collaborations with patients with progressive conditions, it is important to factor in and master methods to maintain engagement and enthusiasm for the project, particularly for long-term initiatives. The process described here is applicable to development of a patient-focused clinical outcome assessment strategy, and specifically of PRO instruments for early-stage Parkinson's. Many of the principles outlined here may be applicable to engaging patients in COA/PRO research in other therapy areas, but context-specific choices would need to be carefully considered throughout the process.

Representation across categories such as race/ethnicity, and other demographics, was not achieved amongst the patient experts, and will be addressed in future collaborations. Although this project was a first step towards international collaboration, future research should include a more diverse representation across countries. The team is working on a shared diversity, equity, and inclusion strategy for future research phases.

## Conclusions

Although additional resources and coordination were required, including patient experts throughout the process had a strong positive impact on PRO instrument design, enabling the development of PRO instruments for early-stage Parkinson’s that better reflect the patient experience when compared with existing instruments. Close collaboration with patients at suitable stages of all future COA research is therefore recommended.

### Supplementary Information

**Additional file 1**: Patient Involvement Plan.

**Additional file 2**: US and UK cultural and language differences identified and addressed by patient experts.

**Additional file 3**: Patient experts’ degrees of meaningful engagement captured using PEIRS-22, by subdomain and research phase. Very high levels of meaningful engagement were achieved for 5 of the 6 patient experts across the two research phases. Overall level of meaningful engagement improved from research phase I to II.

**Additional file 4**: Guidance for reporting involvement of patients and the public.

## Data Availability

The datasets generated during and/or analyzed across both phases of this research are not publicly available as data from non-interventional studies are outside of UCB’s data sharing policy.

## Abbreviations

COA: Clinical outcomes assessment
EUPATI: European Patients’ Academy on Therapeutic Innovation
FDA: Food and Drug Administration
ICH: International Conference on Harmonization
IMI: Innovative Medicines Initiative
MDS-UPDRS: Movement Disorder Society-Unified Parkinson’s Disease Rating Scale
NHC: National Health Council
PCOR: Patient-centered outcomes research
PD: Parkinson’s disease
PDQ-39: Parkinson’s Disease Questionnaire
PEIRS: Patient Engagement in Research Scale
PFDD: Patient-Focused Drug Development
PFMD: Patient Focused Medicines Development
PIP: Patient Involvement Plan
PO: Patient organization
PRO: Patient-reported outcomes
R&D: Research and development
