# Correction to: The value of co-creating a clinical outcome assessment strategy for clinical trial research: process and lessons learnt

**DOI:** 10.1186/s40900-023-00538-y

**Published:** 2024-01-09

**Authors:** Thomas Morel, Karlin Schroeder, Sophie Cleanthous, John Andrejack, Geraldine Blavat, William Brooks, Lesley Gosden, Carroll Siu, Natasha Ratcliffe, Ashley F. Slagle

**Affiliations:** 1 Patient-Centred Outcomes Research, UCB Pharma, Allée de La Recherche 60, 1070 Anderlecht, Brussels, Belgium; 2 Parkinson’s Foundation, New York, NY USA; 3 Modus Outcomes, a Division of Thread, London, UK; 4 Parkinson’s Foundation, New York, NY USA; 5 Parkinson’s UK, London, UK; 6 Aspen Consulting, LLC, Steamboat Springs, CO USA

**Correction to: Research Involvement and Engagement (2023) 9:98** 10.1186/s40900-023-00505-7

Following publication of the original article [1], the authors reported errors in reference 13. The revised link in reference 13 has been indicated hereafter.

The incorrect reference 13 reads:

13. FDA. Assessment of the use of patient experience data in regulatory decision-making final report. https://www.fda.gov/drugs/development-approval-process-drugs/fda-patient-focused-drug-development-guidance-series-enhancing-incorporation-patients-voice-medical. Accessed 06 Mar 2023.

The correct reference 13 should read:

13. FDA. Assessment of the use of patient experience data in regulatory decision-making final report. https://www.fda.gov/drugs/development-approval-process-drugs/assessment-use-patient-experience-data-regulatory-decision-making Accessed 06 Mar 2023.

All the changes requested are implemented in this correction and the original article [1] has been corrected.
