# Supplementary material for: The value of co-creating a clinical outcome assessment strategy for clinical trial research: process and lessons learnt
Source: Res Involv Engagem. 2023 Oct 24;9:98. doi: 10.1186/s40900-023-00505-7 (PMC10598985; doi:10.1186/s40900-023-00505-7)
Supplement: Supplementary file 1 — Additional file 1: Patient Involvement Plan. [file 40900_2023_505_MOESM1_ESM.pdf]

Additional File 1 Patient Involvement Plan

This document describes how the pharmaceutical sponsor (UCB), the Parkinson's Foundation, Parkinson's UK, and people with Parkinson's are working together on clinical outcomes assessment research in early-stage Parkinson's (a clinical outcomes assessment is a tool to measure how a patient feels and functions).

Together, we are equal stakeholders with shared goals. Our ultimate shared goal is to get better therapies to people with Parkinson's more quickly. We want research that is well-designed with strong scientific, evidence-based principles. That research must meet the needs and priorities of the Parkinson's community.

For this project, our goals are to:

- identify what matters most to people with early-stage Parkinson's;
- evaluate current tools to measure Parkinson's;
- determine if there is a need to improve current tools or make better tools and if so;
- improve or create patient-reported outcomes instrument(s).

We are working with external advisors on patient involvement and with clinical experts/research groups (e.g., Michael J. Fox Foundation) where needed. We are striving to work together at as many stages of the research as possible.

We are also striving for new models of patient involvement in this type of research and for co-creation of knowledge.

Patient involvement in clinical outcome assessment research may happen at various research stages, and adopt various levels of intensity. This document identifies and describes opportunities for patient involvement.
