# Supplementary material for: The value of co-creating a clinical outcome assessment strategy for clinical trial research: process and lessons learnt
Source: Res Involv Engagem. 2023 Oct 24;9:98. doi: 10.1186/s40900-023-00505-7 (PMC10598985; doi:10.1186/s40900-023-00505-7)
Supplement: Supplementary file 2 — Additional file 2: US and UK cultural and language differences identified and addressed by patient experts. [file 40900_2023_505_MOESM2_ESM.pdf]

Additional File 2 US and UK cultural and language differences identified and addressed by patient experts

Problematic term or process	Action
**"Doing up a zip"**	"Doing up a zipper" included to align with US English
**"Counting money"**	"Counting paper money" included to reduce ambiguity
**"Difficulty walking outdoors"**	Updated to "difficulty walking on uneven ground" to add context
**Response scales**	Patients' perspective highlighted potential issues; for example, it can be difficult to assess the difference between "very easy" and "easy"
**Instructions for completion of ratings scales**	Patients' perspective highlighted the need for more information; for example, with slowness, the individual should not think about the cause of slowness but focus on speed
**Symptom severity scale**	The scale was separated to ask if symptoms were on the left or right side as this was deemed simplest for patients
