# Supplementary material for: The value of co-creating a clinical outcome assessment strategy for clinical trial research: process and lessons learnt
Source: Res Involv Engagem. 2023 Oct 24;9:98. doi: 10.1186/s40900-023-00505-7 (PMC10598985; doi:10.1186/s40900-023-00505-7)
Supplement: Supplementary file 3 — Additional file 3: Patient experts’ degrees of meaningful engagement captured using PEIRS-22, by subdomain and research phase. Very high levels of meaningful engagement were achieved for 5 of the 6 patient experts across the two research phases. Overall level of meaningful engagement improved from research phase I to II. [file 40900_2023_505_MOESM3_ESM.pdf]

Additional File 3 Patient experts' degrees of meaningful engagement captured using PEIRS-22, by subdomain and research phase. Very high levels of meaningful engagement were achieved for five of the six patient experts across the two research phases. Overall level of meaningful engagement improved from research phase I to II

Phase I

Patient expert #	PEIRS-22 score	Procedural requirements	Convenience	Contributions	Team environment & interaction	Support	Feel valued	Benefits
1	**99***	30.7	13.6	13.6	9.1	9.1	9.1	13.6
2	**89†**	26.1	12.5	11.4	9.1	6.8	9.1	13.6
3	**89†**	29.5	10.2	12.5	9.1	8.0	5.7	13.6
4	**98***	29.5	13.6	13.6	9.1	9.1	9.1	13.6
5	**94***	30.7	13.6	12.5	9.1	9.1	6.8	12.5
6	**80‡**	25.0	10.2	10.2	6.8	6.8	8.0	12.5

Phase II

Patient expert #	PEIRS-22 score	Procedural requirements	Convenience	Contributions	Team environment & interaction	Support	Feel valued	Benefits
1	**100***	31.8	13.6	13.6	9.1	9.1	9.1	13.6
2	**95***	29.5	12.5	12.5	9.1	9.1	9.1	13.6
3	**99***	31.8	12.5	13.6	9.1	9.1	9.1	13.6
4	**98***	31.8	11.4	13.6	9.1	9.1	9.1	13.6
5	**99***	31.8	13.6	13.6	9.1	9.1	9.1	12.5
6	**80‡**	23.9	11.4	10.2	6.8	6.8	8.0	12.5

Degree of meaningfulness: *extremely, †very, ‡moderately. No low degree of meaningfulness was reported

PEIRS Patient Engagement In Research Scale
