# Supplementary material for: The value of co-creating a clinical outcome assessment strategy for clinical trial research: process and lessons learnt
Source: Res Involv Engagem. 2023 Oct 24;9:98. doi: 10.1186/s40900-023-00505-7 (PMC10598985; doi:10.1186/s40900-023-00505-7)
Supplement: Supplementary file 4 — Additional file 4: Guidance for reporting involvement of patients and the public. [file 40900_2023_505_MOESM4_ESM.pdf]

Additional File 4 Guidance for reporting involvement of patients and the public

Section and topic	Item	Reported on page No
**1: Aim**<br><br>**Report the aim of PPI in the study**	This article aims to provide detailed insight into the implementation of a novel process for patient involvement to co-create a COA strategy that more accurately reflects the lived-experience of early-stage Parkinson's and present learnings for future research. The article describes the planning and organizational stages necessary for co-creation, roles performed by patient experts in the multidisciplinary research group throughout the project, and areas where patient experts can provide an impact and value to the output.	3–4
**2: Methods**<br><br>**Provide a clear description of the methods used for PPI in the study**	Six people living with Parkinson's (termed 'patient experts') were involved in the multidisciplinary research team and performed key roles, including qualitative study protocol design, conceptual model development, and subsequent co-creation of two PRO instruments.<br><br>The methods provide details about how patient experts were involved in the study:<br>• Planning patient engagement<br>o Setting up for co-creation: POs were used to recruit the patient experts<br>o Legal and compliance considerations when involving patient experts<br>o How the patient experts were selected<br>o Developing the patient involvement plan to ensure consensus across the multidisciplinary research team<br>o Ensuring the project materials were made as accessible as possible for the patient experts<br>o Highlighting the important considerations that need to be made when including patient experts<br>• Conducting patient engagement in COA/PRO research<br>o Details of the training that patient experts completed<br>o Details of the roles patient experts filled across the different phases of the research<br>o Details of how patient engagement was assessed using the PEIRS	4–13

**3: Study results**<br><br>**Outcomes—Report the results of PPI in the study, including both positive and negative outcomes**	PPI contributed throughout the study from design to data interpretation. The manuscript reports the following results of PPI:<br><br>• Providing feedback on what they found useful and where they would have liked to provide further support<br>• Contributing to the development process<br>• Conceptualizing clinical benefit<br>• Providing input on their direct disease experience<br>• Identifying cardinal concepts in early-stage Parkinson's<br>• Developing the new PRO measures, and the impact on timelines of involving patient experts<br>• Highlighting challenges with patient involvement in COA<br>• Results of the PEIRS assessment	13–17

COA clinical outcomes assessment, *PEIRS* Patient Engagement In Research Scale, *PO* patient organization, *PPI* Patient and Public Involvement, *PRO* patient-reported outcome
